# Supplementary material for: Iodine status of non-pregnant women and availability of food vehicles for fortification with iodine in a remote community in Gulf province, Papua New Guinea
Source: PLoS One. 2019 Nov 15;14(11):e0224229. doi: 10.1371/journal.pone.0224229 (PMC6858069; doi:10.1371/journal.pone.0224229)
Supplement: Attachment — Submitted filename: Review Comments.docx [file pone.0224229.s001.docx]

**Review Comments on**

**“*Iodine Nutrition Status among non-pregnant Women, Awareness of Iodine and Available Food Vehicles for Fortification with Iodine in Kotidanga Rurual LLG, Kerema District, Gulf Province, Papua New Guinea ”***

**PONE-D-19-14979**

**Summary**

Using the survey data collected in a remote area in Gulf Province, Papua New Guinea, this study assessed the iodine status of non-pregnant women, awareness and use of iodized salt, and the availability of other industrially processed foods that maybe fortified with iodine. This study found there was insufficient consumption of adequately iodized salt in this remote area, mainly resulting from low access to and limited consumption of commercial salt due to remoteness, cost, and availability.

**Reviews Comments:**

1. **Sampling**

In lines 127-132, the authors stated that

“*According to the recently released UNICEF Guidance on the Monitoring of Salt Iodization Programmes and Determination of Population Iodine Status [19]“ around 400 urine samples per population group are required to measure the median UIC with 5% precision and 100 urine samples to measure the median UIC with 10% precision”. In the current mini-survey with limited resources, a sample size of 300 non-pregnant women of childbearing age was considered adequate to provide sufficient precision to determine the median UIC.*”

The study surveyed 300 women aged 15 to 45 years old who visited the major markets. Out of 300, 16 observations, who were pregnant women in their first trimester, were excluded from the analysis.

My concerns are as follows:

1) Were the women who visited the major markets systematically different from those who did not visited the markets? If so, how could the authors generalize their results?

2) Reporting error was partly addressed in the study. For example, a subsample (N=62) was randomly selected to check for idolized salt at home among a total of 149 households who reported to have such salt at home. However, there may be a case -- households who reported not to have idolized salt at home actually had such salt. Therefore, the estimates

2. Methods and Analyses

The authors conducted the Shapiro-Wilks test for normality. The result shows that the frequency distribution of the UIC (ug/L) for all women was not normally distributed (p-value = 0.0001) (see Line 313). However, it was not clear whether the authors assume normal distribution for some results in Table 1 as well as in the subsection titled “Comparison of the UIC of non-pregnant women in households with salt and without salt.” If the normal distribution was not assumed, what was assume for the distribution for the statistical analyses?
