# Supplementary material for: Iodine status of non-pregnant women and availability of food vehicles for fortification with iodine in a remote community in Gulf province, Papua New Guinea
Source: PLoS One. 2019 Nov 15;14(11):e0224229. doi: 10.1371/journal.pone.0224229 (PMC6858069; doi:10.1371/journal.pone.0224229)
Supplement: Attachment — Submitted filename: Response to Reviewers.doc [file pone.0224229.s003.doc]

**RESPONSE TO REVIEWERS:**

PLOS ONE Decision: Revision required [PONE-D-19-14979] - [EMID:b5f6943078cd6989]

PONE-D-19-14979

**Title: “*Iodine status of non-pregnant women and availability of food vehicles for fortification with iodine in a remote community in Gulf province, Papua New Guinea*”**

PLOS ONE

Dear Dr Temple,

Thank you for submitting your manuscript to PLOS ONE. After careful consideration, we feel that it has merit but does not fully meet PLOS ONE’s publication criteria as it currently stands. Therefore, we invite you to submit a revised version of the manuscript that addresses the points raised during the review process.

Please consider the revision based on the remaining reviewer's comments.

We would appreciate receiving your revised manuscript by Oct 24 2019 11:59PM. When you are ready to submit your revision, log on to <https://www.editorialmanager.com/pone/> and select the 'Submissions Needing Revision' folder to locate your manuscript file.

If you would like to make changes to your financial disclosure, please include your updated statement in your cover letter.

To enhance the reproducibility of your results, we recommend that if applicable you deposit your laboratory protocols in [protocols.io](http://protocols.io/), where a protocol can be assigned its own identifier (DOI) such that it can be cited independently in the future. For instructions see: [http://journals.plos.org/plosone/s/submission-guidelines#loc-laboratory-protocols](http://journals.plos.org/plosone/s/submission-guidelines#loc-laboratory-protocols)

Please include the following items when submitting your revised manuscript:

- A rebuttal letter that responds to each point raised by the academic editor and reviewer(s). This letter should be uploaded as separate file and labeled 'Response to Reviewers'.
- A marked-up copy of your manuscript that highlights changes made to the original version. This file should be uploaded as separate file and labeled 'Revised Manuscript with Track Changes'.
- An unmarked version of your revised paper without tracked changes. This file should be uploaded as separate file and labeled 'Manuscript'.

Please note while forming your response, if your article is accepted, you may have the opportunity to make the peer review history publicly available. The record will include editor decision letters (with reviews) and your responses to reviewer comments. If eligible, we will contact you to opt in or out.

We look forward to receiving your revised manuscript.
Kind regards,

Marly A. Cardoso, Ph.D.
Academic Editor
PLOS ONE


[Note: HTML markup is below. Please do not edit.]

Reviewers' comments:

Reviewer's Responses to Questions

**Comments to the Author:**
1. If the authors have adequately addressed your comments raised in a previous round of review and you feel that this manuscript is now acceptable for publication, you may indicate that here to bypass the “Comments to the Author” section, enter your conflict of interest statement in the “Confidential to Editor” section, and submit your "Accept" recommendation.

Reviewer #1: All comments have been addressed

Reviewer #3: (No Response)

**Response:**

Many thanks for this feedback.

2. Is the manuscript technically sound, and do the data support the conclusions?

The manuscript must describe a technically sound piece of scientific research with data that supports the conclusions. Experiments must have been conducted rigorously, with appropriate controls, replication, and sample sizes. The conclusions must be drawn appropriately based on the data presented. 

Reviewer #1: Yes

Reviewer #3: Yes

**Response:**

Many thanks for this feedback.

3. Has the statistical analysis been performed appropriately and rigorously? 

Reviewer #1: Yes

Reviewer #3: Yes

**Response:**

Many thanks for this feedback.

4. Have the authors made all data underlying the findings in their manuscript fully available?

The [PLOS Data policy](http://www.plosone.org/static/policies.action#sharing) requires authors to make all data underlying the findings described in their manuscript fully available without restriction, with rare exception (please refer to the Data Availability Statement in the manuscript PDF file). The data should be provided as part of the manuscript or its supporting information, or deposited to a public repository. For example, in addition to summary statistics, the data points behind means, medians and variance measures should be available. If there are restrictions on publicly sharing data—e.g. participant privacy or use of data from a third party—those must be specified.

Reviewer #1: Yes

Reviewer #3: Yes

**Response:**

Many thanks for this feedback.

5. Is the manuscript presented in an intelligible fashion and written in standard English?

PLOS ONE does not copyedit accepted manuscripts, so the language in submitted articles must be clear, correct, and unambiguous. Any typographical or grammatical errors should be corrected at revision, so please note any specific errors here.

Reviewer #1: Yes

Reviewer #3: Yes

**Response:**

Many thanks for this feedback.

6. Review Comments to the Author

Please use the space provided to explain your answers to the questions above. You may also include additional comments for the author, including concerns about dual publication, research ethics, or publication ethics. (Please upload your review as an attachment if it exceeds 20,000 characters)

Reviewer #1: All the suggested changes have been addressed to a satisfactorily standard. I have no further comments.

Reviewer #3: The authors have addressed most of my first round comments adequately. Thank you for attention to the details of my comments and those of the other reviewers. I have a few remaining comments.

1. Lines 149-155 (and in reference to results presented in lines 276-286, and also 398-402). I note that all markets had the one brand of commercial salt that was then analysed for iodine content and comparison. Because all of the salt from a single brand was likely produced in the same facility (or at least under common company guidance), it may be useful to provide an additional sentence on the market share or importance of this brand. Although recognizing the market regulation, one might expect that more variation exists across brands than within a single brand. This concern is of less importance if this brand represents a major share of salt sold commercially in the country (or survey region).

**Response**:

We agree with the observation of reviewer #3. However, we cannot comment on the market share of this brand of salt or the importance of this brand. We can only state that at the time of this study only one brand of salt was available in the market, unlike an earlier study in which three different brands of salt were available in the markets (see reference below). As already stated, the study site is a remote inaccessible mountainous area without infrastructure, and there is no road access. The way of getting there is mainly by walking along mountain paths or by air (helicopter) transport, which is very expensive. It usually takes about 3 days walking during daylight from the location of the hospital to the closest settlement in Kerema, Gulf province or Menyamya in Morobe province. This may imply that the various commercial items including brand(s) of salt in the markets depend on what the traders were able to purchase and at what cost and how fast they are able to make profit from the sale of their products.

*Ref: “Goris J, Temple V, Zomerdijk N, Codling K. Iodine status of children and knowledge, attitude, practice of iodised salt use in a remote community in Kerema district, Gulf province, Papua New Guinea. PLoS ONE. 2018; 13(11):e0197647. doi: https://doi.org/10.1371/journal.pone.0197647”*

2. Table 3. I agree with a previous comment about the length of Table 3. One possible approach to making the length more manageable would be to break the table into 2, with Q14 and following questions in a second table. The second table with focus on the use of “other processed foods” (as described line lines 351-356).

**Response:**

As suggested by reviewer #3 we have divided Table 3 into two separate tables, with Table 3 covering questions 1 to 13, including responses on use of salty flavourings and commercial salt and Table 4, covering questions 14 to 23, including responses to the use of industrially processed foods.

Table 3: Responses on use of salty flavourings and, commercial salt

Table 4: Responses to use of industrially processed foods

3. Lines 453-456. Related to previous comment #7. Thank you for being more careful throughout about the information on “awareness” that had been previously emphasized in title and discussion. To be more precise here, I suggest rewording line 454-455 to read: “Low education level and remoteness may contribute [i.e., not “may have contributed”] to the apparent lack of awareness…” That makes more clear that there is no finding in the analysis presented on “awareness” per se.

**Response:**

We have changed the text as suggested by reviewer #3.

“Low education level and remoteness may contribute to the apparent lack of awareness of the need to consume adequate amounts of iodine for optimal growth and development ………”

Suggested edits:
Line 36: change to: “Salt was available on the interview day…”
Line 67: change to: “Salt iodisation has been implemented…”
Lines 77-78: change to: “…38% of household had no salt in the household, and women in these households had lower…”
Lin 486: drop comma to read: “…would improve iodine intake if adequate and sustainable…” This is an essential condition to the statement.

**Response:**

We have made all the edits in the text (line 36, line 67, lines 77-78 and line 486) as suggested by reviewer #3.

7. PLOS authors have the option to publish the peer review history of their article ([what does this mean?](https://journals.plos.org/plosone/s/editorial-and-peer-review-process#loc-peer-review-history)). If published, this will include your full peer review and any attached files.

If you choose “no”, your identity will remain anonymous but your review may still be made public.

**Do you want your identity to be public for this peer review?** For information about this choice, including consent withdrawal, please see our [Privacy Policy](https://www.plos.org/privacy-policy).

Reviewer #1: No
Reviewer #3: No

[NOTE: If reviewer comments were submitted as an attachment file, they will be attached to this email and accessible via the submission site. Please log into your account, locate the manuscript record, and check for the action link "View Attachments". If this link does not appear, there are no attachment files to be viewed.]

While revising your submission, please upload your figure files to the Preflight Analysis and Conversion Engine (PACE) digital diagnostic tool, <https://pacev2.apexcovantage.com/>. PACE helps ensure that figures meet PLOS requirements. To use PACE, you must first register as a user. Registration is free. Then, login and navigate to the UPLOAD tab, where you will find detailed instructions on how to use the tool. If you encounter any issues or have any questions when using PACE, please email us at [figures@plos.org](mailto:figures@plos.org). Please note that Supporting Information files do not need this step.

*In compliance with data protection regulations, you may request that we remove your personal registration details at any time.*  [*(Remove my information/details)*](https://www.editorialmanager.com/pone/login.asp?a=r)*. Please contact the publication office if you have any questions.*
